# Apremilast in the Management of Disseminated Granuloma Annulare

**DOI:** 10.7759/cureus.14918

**Published:** 2021-05-09

**Authors:** Tejas P Joshi, Jaime Tschen

**Affiliations:** 1 Dermatology, Baylor College of Medicine, Houston, USA; 2 Dermatology, St. Joseph Dermatopathology, Houston, USA

**Keywords:** granuloma annulare, disseminated granuloma annulare, apremilast, off-label drug use, psoriasis

## Abstract

Granuloma annulare (GA) is a common inflammatory skin condition that manifests as annular skin colored to erythematous papules and plaques. Disseminated GA is a subtype of GA that presents with diffuse cutaneous involvement. While topical and intralesional corticosteroids and phototherapy have been used as therapies for GA, there is no consensus on the best course of treatment for GA. Apremilast is a phosphodiesterase 4 (PDE4) inhibitor that has been Food and Drug Administration (FDA) approved for psoriasis, psoriatic arthritis, and oral ulcers associated with Behcet’s disease; apremilast has also shown promise off-label for other inflammatory skin conditions. Here, we present the case of a woman in whom apremilast use led to an almost complete resolution of her disseminated GA. Our patient tolerated apremilast well and reported no side effects. We also review the literature on the use of apremilast in other patients with GA.

## Introduction

Granuloma annulare (GA) is a granulomatous skin condition that presents as skin colored to erythematous papules and plaques. Disseminated GA is a subtype of GA with diffuse cutaneous involvement. While the precise etiology of GA remains poorly understood, a Th1 mediated delayed hypersensitivity reaction has been implicated in the development of GA. Histologically, GA is characterized by the presence of palisading histiocytes, degraded collagen, and mucin [1]. Topical corticosteroids or intralesional triamcinolone are considered to be first line therapies for local disease. While phototherapy has shown to be effective for generalized disease, there nevertheless exists no established paradigm of care for disseminated GA [2].

Apremilast is a phosphodiesterase 4 (PDE4) inhibitor that has been Food and Drug Administration (FDA) approved for psoriasis, psoriatic arthritis, and oral ulcers associated with Behcet’s disease [3]. As apremilast downregulates cytokines central to the pathogenesis of many inflammatory skin conditions, it has been indicated for off-label use in alopecia areata, atopic dermatitis, cutaneous sarcoidosis, discoid lupus erythematosus, hidradenitis suppurativa, and lichen planus [4]. Here, we report the case of a 77-year-old woman in whom apremilast treatment led to an almost complete resolution of her disseminated GA.

## Case presentation

A 77-year-old woman with a history of plaque psoriasis and arthritis presented for evaluation of a burning body rash and plaques on her face and upper extremities. Her medications include levothyroxine and escitalopram. She is allergic to ciprofloxacin, metoprolol, and sulfa drugs. Her past medical history was positive for oral lichen planus. She did not have a relevant family history of plaque psoriasis or disseminated GA.

**Figure 1:**
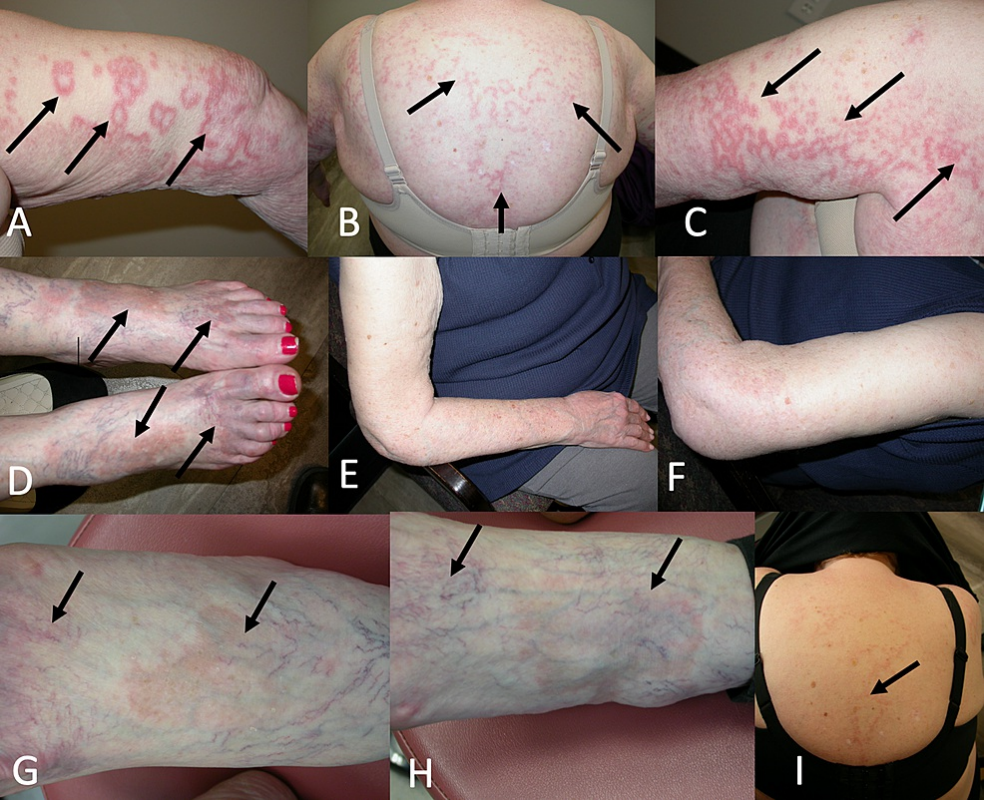
(A) Granuloma annulare (GA) on the right arm at initial evaluation. Spread of GA to back (B), left arm (C), and feet (D) at three-month follow-up. Regression of GA lesions on right (E) and left (F) arms at six-month follow-up. Regression of GA on bilateral feet (G) and (H) at seven-month follow-up. (I) Slight flare-up of GA observed on back at seven-month follow-up.

**Figure 2:**
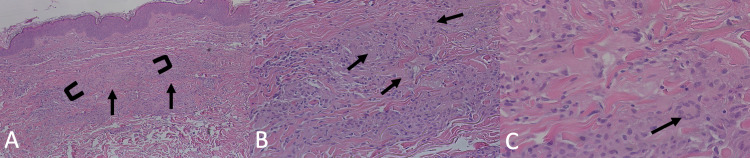
Histopathology of punch biopsy from patient’s right arm displaying characteristic features of granuloma annulare: (A) bracket indicates palisading histiocytes and arrows indicate degraded collagen; 40x magnification (B) arrows point towards regions of necrobiosis; 100x magnification (C) arrow points to multi-nucleated giant cell; 200x magnification.

Physical examination revealed plaques on her feet, face, and arms that were erythematous, annular, circinate, and non-scaly, as well as a cutaneous horn with an irritated seborrheic keratosis at the base on her right wrist (Figure 1A). The patient’s skin examination was otherwise unremarkable. A 4-mm punch biopsy of the lesion on her right arm revealed granulomatous inflammation with areas of necrobiosis consistent with GA (Figure 2). The patient was started on apremilast 30 mg twice a day. Apart from apremilast, she did not use topical steroids. At three-month follow-up, the GA was found to have spread to her chest, back, left upper extremity, and feet (Figures 1B-1D). Apremilast treatment was continued, and at six-month follow-up, the lesions on the chest and bilateral upper extremities had regressed entirely (Figures 1E-1F). The lesions on her feet improved as well but did not resolve completely. At seven-month follow-up, the patient’s remission was mostly durable, with no new lesions appearing over the upper extremities; furthermore, the GA on the patient’s feet substantially regressed (Figures 1G-1H). Yet, a mild flare-up was noted on the patient’s cervical to lumbar back (Figure 1I). The patient’s skin examination was otherwise unremarkable. Also noteworthy, the patient’s burning sensation from the rash disappeared shortly after starting apremilast, and at seven-month follow-up, reported only slight pruritus over the flare-up on her back. The patient denied any gastrointestinal side effects and tolerated apremilast well. The patient was instructed to continue with apremilast.

## Discussion

GA was first described in 1895 by Colcott-Fox as a “ringed eruption”[5]. Classically, GA manifests as annular skin colored to erythematous papules and plaques. Disseminated GA is a variant of GA with more generalized cutaneous involvement. While a Type IV hypersensitivity reaction has been implicated as the mechanism underlying GA pathogenesis, the precise etiology of GA eludes facile explanation. GA has been linked, albeit tenuously, to diabetes mellitus, malignancy, thyroid disease, and dyslipidemias [1]. There is a paucity of literature that offers any consensus on the best course of treatment for GA. Wang and Khachemoune reviewed the treatments for GA and report a wide gamut of treatments to have been used for GA [2]. Nevertheless, some of these treatments have concerning side effect profiles. Chlorambucil, for instance, has been reported in the management of GA [6] yet it is a very risky treatment as it can precipitate hemorrhagic cystitis. Similarly, dapsone was recently reported to be used for the treatment of GA in 26 patients, and while 54% of patients experienced improvement, 31% of patients experienced myelosuppression, which required treatment to be discontinued [7].

Apremilast has been indicated as a treatment for psoriasis, psoriatic arthritis, and oral ulcers associated with Behcet’s disease [3]. Apremilast works by inhibiting PDE4, thereby preventing cyclic adenosine monophosphate (cAMP) hydrolysis; elevated cytosolic cAMP levels then work to dampen the production of inflammatory cytokines (tumor necrosis factor-alpha (TNF-α), interleukin (IL)-6, IL-12, and interferon-γ (IFN-γ)) important in psoriasis pathogenesis. As TNF-α, IL-6, and IL-12 have also been implicated in GA pathogenesis [1], it is mechanistically rational to consider the therapeutic potential of apremilast in the management of GA. Furthermore, apremilast has a mild side effect profile, with mild to moderate gastrointestinal symptoms that typically resolve with continued treatment [8].

In our literature review, we identified two case series reporting the off-label use of apremilast in the management of GA. Blum and Altman reported two cases in which apremilast was effective for the treatment of GA refractory to steroids. One patient experienced partial resolution of their lesions and the other patient experienced an improvement in erythema and lesion induration. Both patients tolerated apremilast well [9]. Bishnoi et al. reported apremilast to be effective in the treatment of recalcitrant GA in four additional patients. Three patients reported a reduction in lesion number and one patient reported decreased erythema and pruritis. Apremilast was generally well tolerated, with one patient reporting mild gastrointestinal disturbance and myalgia and another patient reporting nausea and myalgia. The other two patients did not experience any adverse effects [10].

Although spontaneous improvement of GA in our patient cannot be completely excluded, the temporal clinical and symptomatic improvement makes it likely that the patient’s remission is putatively due to apremilast treatment. The minor flare-up observed at seven-month follow-up is similar to what is reported in the literature in other patients who experienced improvement without complete resolution [9,10]. Furthermore, we note that while spontaneous remission for localized GA is common, the more generalized form of the disease, which was present in our patient, can persist for years [11].

## Conclusions

GA is a common skin condition with no well-established paradigm of care. Our patient experienced near-complete resolution of GA in response to apremilast treatment. At seven-month follow-up, she is tolerating apremilast well and denies any side effects. Six other cases in the literature demonstrate that apremilast can be a safe and efficacious treatment for GA. Altogether, our case further supports the notion that apremilast may have a seminal application in the management of GA.
